# Medizinischer Kinderschutz während des Corona-Lockdowns: Vergleichende Befunde der Kinderschutzfälle aus Kliniken und Ambulanzen in Deutschland

**DOI:** 10.1007/s00112-021-01135-7

**Published:** 2021-02-16

**Authors:** T. Heimann, J. Ewert, F. Metzner, F. Sigmund, A. Jud, S. Pawils

**Affiliations:** 1 Institut für Rechtsmedizin, Universitätsklinikum Hamburg-Eppendorf, Hamburg, Deutschland; 2 Klinik und Poliklinik für Kinder- und Jugendmedizin, Universitätsklinikum Hamburg-Eppendorf, Hamburg, Deutschland; 3 Institut und Poliklinik für Medizinische Psychologie, Universitätsklinikum Hamburg-Eppendorf, Martinistraße 52, W26, 20246 Hamburg, Deutschland; 4 Klinik für Kinder- und Jugendpsychiatrie/Psychotherapie, Universitätsklinikum Ulm, Ulm, Deutschland; 5 Soziale Arbeit, Hochschule Luzern, Luzern, Schweiz

**Keywords:** Kindesmisshandlung, Vernachlässigung, Dunkelziffer, COVID-19, „Social distancing“, Child abuse and neglect, COVID-19, Lockdown, Social distancing, Unreported cases

## Abstract

**Hintergrund:**

Es gibt Anhaltspunkte dafür, dass die Gefahr von Kindesmisshandlung, sexuellem Kindesmissbrauch und Vernachlässigung während der strengen Kontaktbeschränkungen im pandemiebedingten Lockdown zugenommen hat, während der Wegfall der gewohnten Mechanismen zur sozialen Kontrolle mutmaßlich zu einer Zunahme des Dunkelfelds geführt hat.

**Ziel der Arbeit:**

Anhand der Kinderschutzfallzahlen und -qualitäten deutscher Kinderkliniken und Kinderschutzambulanzen sollen Aussagen über Häufigkeit und Schwere vermuteter Kindeswohlgefährdung während des pandemiebedingten Lockdowns getroffen werden.

**Methoden:**

Im Mai 2020 erfolgte eine Onlinebefragung mit Items a) zur Beschreibung der Institution, b) zur nach Alter, Gewaltform und Schweregrad differenzierten Anzahl von Kinderschutzfällen in März/April 2019 und 2020 sowie c) zu Besonderheiten und Ideen für den Kinderschutz während der Pandemie.

**Ergebnisse:**

In einer Vollerhebung wurden 343 Kinderkliniken und medizinische Kinderschutzambulanzen zur Onlinebefragung eingeladen; die Teilnahmequote lag bei 46 %. Es gaben 81 Einrichtungen Gesamtfallzahlen für März/April 2019 und März/April 2020 an. Bei den Ambulanzen konnte ein Rückgang von 454 auf 387 Fälle (−15 %) verzeichnet werden, bei den Kinderschutzgruppen von 307 auf 246 (−20 %). Hinsichtlich der Altersgruppen und der Formen der Gefährdung fanden sich keine signifikanten Unterschiede.

**Schlussfolgerung:**

Die Untersuchung beschreibt einen Rückgang der absoluten Fallzahlen im medizinischen Kinderschutz während des Lockdowns im März und im April 2020. Dieses Ergebnis stützt die Vermutung, dass das Dunkelfeld gefährdeter Kinder weiter gestiegen sein könnte. Weitere Datenerhebungen nach dem Lockdown werden die längerfristigen Auswirkungen besser bewerten können.

## Abstract (en)

**Background:**

There are indications that during the lockdown and the measures of social distancing due to the coronavirus disease 2019 (COVID-19) pandemic, physical and sexual child abuse and neglect may have increased. Additionally, decreased social control may have led to a lower detection rate of child protection cases.

**Objective:**

This study aimed to better understand the impact of the lockdown in Germany on the frequency and severity of child abuse and neglect. To do so the quantity and quality of cases of suspected child abuse and neglect in German pediatric departments and outpatient departments for medical child protection were examined.

**Methods:**

In May 2020 a total of 343 institutions of medical child protection were invited to fill in a questionnaire with items describing their institution and items depicting cases of child protection in March and April 2019 and 2020 regarding age, form and severity of abuse as well as items to describe particular remarks and ideas for child protection during the pandemic.

**Results:**

Out of the 343 pediatric departments and outpatient departments of medical child protection invited, the participation rate was 46%. In this study 81 institutions reported the total cases of suspected child abuse or neglect for both March and April 2019 and 2020. The number of cases dropped from 454 to 387 (−15%) in outpatient child abuse clinics and from 307 to 246 (−20%) in pediatric inpatient departments. Regarding the age of affected children and the form of abuse no significant differences were found.

**Conclusion:**

The study found a decrease in reported cases of suspected child abuse and neglect during the lockdown in March and April 2020 compared to 2019. While the results do not show an increase of total child abuse and neglect, as suspected by many professionals, the decrease in reported cases may be explained by a steep increase in unreported cases due to reduced social control.

Während der COVID-19-Pandemie werden weltweit Einschränkungen sozialer Kontakte durchgesetzt. Deutschlandweit traten die weitreichendsten Maßnahmen, der sog. Lockdown, am 16.03.2020 in Kraft. Die Gefahr steigender Zahlen von Kindesmisshandlung, -missbrauch und -vernachlässigung wurde medial und in Fachkreisen schon früh diskutiert. Der folgende Beitrag gibt die Ergebnisse einer Umfrage an allen deutschen Kinderkliniken und Kinderschutzambulanzen bezüglich der Fallzahlen, -schwere und -qualität während des Lockdowns wieder.

## Steigender Stress für Familien

Kinder und Jugendliche sind keine Risikogruppe für schwere Verläufe der Coronavirus-Krankheit-2019 (COVID-19) [17], aber waren durch die Schließung von Kitas, Schulen und Freizeitangeboten sowie durch minimierte Kontakte in besonderem Maß vom Lockdown betroffen. Das eingeschränkte Angebot der Jugend- und Familienhilfe stellte besonders für belastete Familien eine Herausforderung dar [9, 26]. Kindern fehlte es an Möglichkeiten, sich Entlastung, Beratung oder außerfamiliären Schutz zu suchen [24]. In vielen Familien kam es zu Konflikten; fast jedes vierte Kind fühlte sich einsam [15]. Die Inanspruchnahme von telefonischen Beratungsstellen für Kinder und Eltern erhöhte sich [27].

In Krisen aufgrund von Naturkatastrophen oder Wirtschaftsrezession stieg in der Vergangenheit das Risiko für Kindesmisshandlung häufig über das Ende der Krisen hinaus an [2, 6, 11, 25]. Befürchtet wird daher, dass sich das Misshandlungsrisiko insbesondere in den Familien, die bereits vor der COVID-19-Pandemie sehr belastet waren, während des Lockdowns erhöhte [4, 5], insbesondere wenn von Gewalt betroffene Kinder in der zum Schutz der Bevölkerung verordneten Kontaktsperre mit den TäterInnen zusammenlebten [7].

## Wegfall sozialer Kontrolle

Während die Gefahr für Misshandlungen in den Familien anstieg, entfielen gleichzeitig wichtige Mechanismen der sozialen Kontrolle. Verletzungen oder Verhaltensänderungen, die sonst in der Kita oder Schule aufgefallen wären, könnten während des Lockdowns häufig unentdeckt geblieben sein. Betroffenen Kindern fehlten wichtige AnsprechpartnerInnen, um sich anzuvertrauen und Übergriffe zu melden. Während Kinder im Schulalter häufig noch mit ihren LehrerInnen in Kontakt standen, sahen Kleinkinder und Kinder insbesondere aus sozioökonomisch benachteiligten Familien u. U. über Wochen nur ihre engsten Familienmitglieder. Dementsprechend verzeichneten etwa 25 % der deutschen Jugendämter in diesem Zeitraum einen Rückgang an Gefährdungsmeldungen [16]. Vermutet werden muss, dass die Isolationsmaßnahmen nicht nur vulnerable Kinder aus Familien mit hoher Stressbelastung oder in prekären Lebensverhältnissen gefährdeten, sondern auch Kinder, die bis dahin nicht zur Risikogruppe für Kindeswohlgefährdung gehörten.

## Mangel an empirisch fundiertem Wissen

Weltweit wurde von steigenden Zahlen häuslicher Gewalt v. a. gegen Frauen berichtet, die sich bislang v. a. aus einer Zunahme lokaler Polizeieinsätze diesbezüglich sowie steigenden Anrufzahlen bei Hilfetelefonen ableiten [3, 14, 18]. Über die Häufigkeit von Gewalt gegen Kinder in diesem Zeitraum ist ebenfalls wenig bekannt. In einer bevölkerungsrepräsentativen Umfrage in Deutschland gaben etwa 7 % der befragten Frauen an, dass mindestens eins ihrer Kinder während des Lockdowns Opfer von körperlicher Gewalt geworden ist [23]. Während die Zahlen erwachsener PatientInnen in Krankenhäusern bezüglich lebensbedrohlicher Krankheiten während des Lockdowns zurückgingen [19], zeigten anekdotische Berichte steigende Zahlen von in Krankenhäusern versorgten nichtakzidentellen Schädel-Hirn-Traumata auf [21]. Die Medizinische Kinderschutzhotline verzeichnete nach einem Rückgang der Anrufe in den Monaten März und April ab Mai 2020 einen Anstieg der Anrufe über das Vorjahresniveau hinaus [1].

## Medizinischer Kinderschutz in Deutschland

Die deutschen Jugendämter führten 2019 etwa 6 % ihrer Gefährdungseinschätzungen nach Meldungen von Kinderschutzfällen aus dem Gesundheitswesen durch [22]. Da in Deutschland keine Meldepflicht für Fälle von vermuteter Kindeswohlgefährdung besteht, ist von einer hohen Zahl nichtgemeldeter und daher nichtregistrierter Kinderschutzfälle, die in der medizinischen Versorgung aufgefallen sind, auszugehen. Nachdem die Deutsche Gesellschaft für Kinderschutz in der Medizin (DGKiM) und die Deutsche Akademie für Kinder- und Jugendmedizin (DAKJ) ein strukturiertes und multiprofessionelles Vorgehen im medizinischen Kinderschutz gefordert haben, steigt die Zahl der Kinderschutzambulanzen und -gruppen kontinuierlich an [20]. Ein Register und eine einheitliche Dokumentation von Kinderschutzfällen fehlen in Deutschland jedoch bisher. Die Abrechnung der Diagnostik bei Verdacht auf Kindeswohlgefährdung ist seit 2018 über eine Aufnahme der OPS-Kinderschutzprozedur 1‑945 in den Fallpauschalenkatalog möglich. Eine spezifische Verschlüsselung ist über das Klassifikationssystem ICD-10 zwar möglich, wird aber wenig genutzt, sodass die Anzahl der Kinder, die aufgrund von Misshandlung oder Vernachlässigung medizinisch versorgt wurden, nicht den Abrechnungsdaten der Krankenkassen entnommen werden kann. Ziel der vorliegenden Untersuchung war es, anhand der in deutschen Kinderkliniken und Kinderschutzambulanzen dokumentierten Kinderschutzfälle während der ersten 2 Monate des pandemiebedingten Lockdowns Aussagen über die Häufigkeit und Schwere von Gewalt gegen Kinder während des Lockdowns zu treffen.

## Methodik

Mittels eines Onlinesurveys wurden in einer Vollerhebung deutschlandweit kinderversorgende Kliniken und Ambulanzen zur Entwicklung der Kinderschutzfälle während des COVID-19-Lockdowns befragt.

### Stichprobe

Eingeladen wurden 365 kinderversorgende Kliniken und Ambulanzen in Deutschland, die im Mai 2020 auf der Homepage oder im E‑Mail-Verteiler der DGKiM eingetragen waren. Aufgrund fehlerhafter Kontaktdaten wurden 22 Einrichtungen nicht erreicht. Von den 343 erreichten Kliniken und Ambulanzen nahmen 159 Einrichtungen mit insgesamt 188 Versorgungsbereichen an der Befragung teil, sodass eine Teilnahmequote von 46 % erreicht wurde.

### Instrumente

Zur Befragung wurden Online-Fragebogen mithilfe der Computer-Software SoSci Survey erstellt. Diese enthielten 13 selbstentwickelte Items zur Beschreibung der Einrichtung, zur nach Alter, Gewaltform und Schweregrad differenzierten Anzahl von Kinderschutzfällen im März und im April in den Jahren 2019 und 2020, zu Besonderheiten sowie zu Ideen und Wünschen für den Kinderschutz während der COVID-19-Pandemie. Die Gewaltformen wurden differenziert nach etablierten Unterformen erfasst, wobei angesichts der unterschiedlichen Dokumentationssysteme auf Operationalisierungen der Kategorien verzichtet wurde. Der Schweregrad wurde abgefragt als Fälle, die schon allein aufgrund der Schwere auftretender Verletzungen stationärer Behandlung bedurft hätten. Die Fragen waren über Multiple-Choice-, dichotome oder freitextliche Antwortformate zu beantworten. Die durchschnittliche Bearbeitungsdauer der Fragebogen ließ sich nicht ermitteln, da die Befragung zur Recherche der Fallzahlen unterbrochen werden konnte.

### Durchführung

Zur Prüfung von Expertenvalidität und Praktikabilität des Fragebogens wurde ein Pretest durchgeführt, in dem 4 Kliniken und Ambulanzen sowie Mitglieder der DGKiM Änderungen hinsichtlich des Inhalts und Formats vorschlugen. Die ethische Unbedenklichkeit der Studie wurde von der lokalen psychologischen Ethikkommission am Zentrum für Psychosoziale Medizin am UKE bestätigt (LPEK-0158). Die Kliniken und Ambulanzen wurden im Mai 2020 über die DGKiM zur Teilnahme an der Befragung eingeladen und nach 4 Wochen an die Befragung erinnert. Die Einrichtungen erhielten kein Incentive für die Teilnahme an der Befragung.

### Statistische Analyse

Zum Vergleich der Fallzahlen zwischen 2019 und 2020 wurden deskriptive Verteilungsmaße wie Median (Md), Range sowie kumulierte absolute Häufigkeiten berechnet. Um Unterschiede auf Signifikanz zu prüfen, wurden Wilcoxon-Tests berechnet. In die Wilcoxon-Tests wurden alle Einrichtungen einbezogen, die sowohl Angaben zu 2019 wie auch 2020 machten.

## Ergebnisse

### Beschreibung der teilnehmenden Einrichtungen

Von den *n* = 159 teilnehmenden Kliniken und Ambulanzen aus allen 16 Bundesländern boten *n* = 120 (75 %) stationäre und *n* = 68 (43 %) ambulante Versorgung im Bereich des medizinischen Kinderschutzes an; *n* = 29 Einrichtungen (18 %) boten sowohl ambulante als auch stationäre Versorgung an (Tab. 1).

**Table 1:** 

	Ambulante Kinderschutzeinrichtungen (*n* = 68)	Stationäre Kinderschutzeinrichtungen (*n* = 120)
	*n (%)*	*n (%)*
*Region*		
Alte Bundesländer	52 (77 %)	78 (65 %)
Neue Bundesländer (inkl. Berlin)	16 (24 %)	42 (35 %)
*Gründungsjahr*		
2011–2020	31 (58 %)	59 (71 %)
2001–2010	15 (28 %)	23 (28 %)
1991–2000	4 (8 %)	1 (1 %)
1981–1990	3 (6 %)	–
*Kinderschutzfälle pro Jahr*^a^		
Range (Median)/MW	0–1231 (44)/122	0–348 (10)/28

^a^Die Fallzahlen beziehen sich auf das Jahr 2019

Die Hälfte der Kinderschutzambulanzen hatte 2019 mehr als 44 Kinderschutzfälle dokumentiert, während die Hälfte der stationären Einrichtungen mehr als 10 Kinderschutzfälle versorgt hatte.

### Anzahl und Art der Kinderschutzfälle im Überblick

Die Einrichtungen berichteten für März und April 2019 insgesamt *n* = 1118 Kinderschutzfälle (Tab. 2). Im März und im April 2020 wurden insgesamt *n* = 702 Kinderschutzfälle dokumentiert (−37 %). In beiden Zeiträumen zeigte sich ein Range von 0 bis 262 Fällen pro Einrichtung. Der deskriptive Vergleich nur der Einrichtungen, die ihre Fallzahlen für beide Zeiträume angaben, ergab zwischen 2019 und 2020 eine Verringerung um *n* = 67 (−15 %) der Kinderschutzfälle im ambulanten bzw. um *n* = 61 (−20 %) im stationären Bereich.

**Table 2:** 

Kinderschutzfälle	Ambulante Kinderschutzeinrichtungen (*n* = 68)		Stationäre Kinderschutzeinrichtungen (*n* = 120)	
	März/April 2019	März/April 2020	März/April 2019	März/April 2020
	*n Fälle [Range]* *(n Einrichtungen)*	*n Fälle [Range]* *(n Einrichtungen)*	*n Fälle* *[Range]* *(n Einrichtungen)*	*n Fälle [Range]* *(n Einrichtungen)*
**Gesamt**	**769** [0–262] (34)	**430** [0–81] (28)	**349** [0–53] (64)	**272** [0–42] (61)
Gesamt der Einrichtungen mit vollständigen Datensätzen (*n* = 81)	**454** [0–156] (27)	**387** [0–81] (27)	**307** [0–53] (54)	**246** [0–42] (54)
*Alter der Patienten*^*a*^				
< 1 Jahr	**34** [0–11] (24)	**43** [0–10] (22)	**97** [0–20] (47)	**77** [0–11] (47)
1–2 Jahre	**49** [0–12] (25)	**47** [0–7] (24)	**38** [0–5] (46)	**45** [0–11] (46)
3–5 Jahre	**90** [0–18] (26)	**96** [0–27] (24)	**35** [0–5] (48)	**28** [0–3] (46)
6–11 Jahre	**82** [0–23] (27)	**83** [0–18] (24)	**33** [0–9] (46)	**36** [0–5] (49)
12–17 Jahre	**71** [0–12] (27)	**49** [0–9] (23)	**64** [0–16] (45)	**70** [0–18] (46)
*Gewaltformen*^a^				
Misshandlung	**241** [0–31] (30)	**217** [0–26] (25)	**136** [0–19] (45)	**103** [0–9] (47)
Vernachlässigung	**64** [0–23] (26)	**52** [0–25] (22)	**83** [0–15] (42)	**92** [0–14] (43)
Sexueller Missbrauch	**82** [0–19] (29)	**79** [0–37] (24)	**13** [0–2] (42)	**22** [0–4] (42)
Stationärer Behandlungsbedarf, unspezifisch	**72** [0–25] (26)	**86** [0–34] (21)	–	–
Schütteltrauma	–	–	**7** [0–1] (42)	**9** [0–3] (43)
Stationärer Behandlungsbedarf (exkl. Schütteltrauma)	–	–	**78** [0–26] (40)	**78** [0–16] (39)

^a^Nicht alle Einrichtungen konnten detaillierte Angaben machen

Die Häufigkeit der Fälle in den unterschiedlichen Altersklassen unterschied sich zwischen beiden Erhebungszeiträumen. Der größte Unterschied wurde für die Altersklasse der 12- bis 17-Jährigen gefunden, für die 2020 *n* = 16 Kinderschutzfälle weniger als 2019 dokumentiert wurden. Für die am häufigsten dokumentierte Gewaltform „Misshandlung“ wurden während des Lockdowns 2020 *n* = 57 Fälle weniger als im Vergleichszeitraum 2019 registriert. Alle anderen Fallzahlen unterschieden sich im zeitlichen Vergleich marginal.

**Figure 1:**
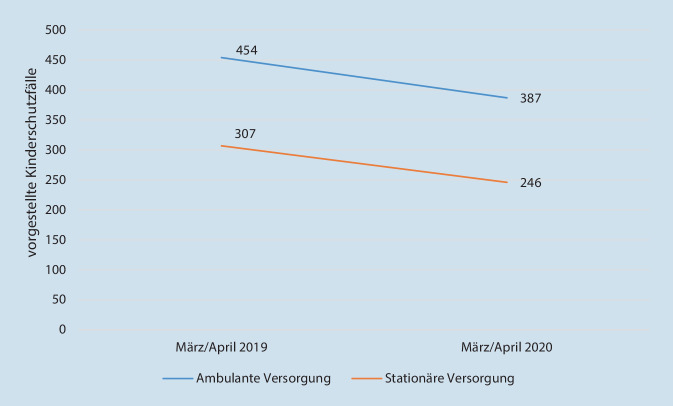

Hinsichtlich der Anzahl an dokumentierten Kinderschutzfällen, Altersgruppen und Misshandlungsformen in den Vergleichszeiträumen 2019 und 2020 zeigten sich keine signifikanten Unterschiede (Abb. 1).

### Besonderheiten im medizinischen Kinderschutz während des Lockdowns

Von den 159 Institutionen haben 78 Einrichtungen freitextliche Angaben gemacht. Etwa 31 % der Kliniken und Ambulanzen (*n* = 24) meldeten Veränderungen in den Fallzuweisungen während des Lockdowns zurück. Rund 12 % der Institutionen (*n* = 9) hatten weniger Zuweisungen durch die Jugendämter verzeichnet. Etwa 13 % der befragten Einrichtungen (*n* = 10) berichteten von Besonderheiten in Bezug auf die Vorstellungsgründe, zu denen vorrangig elterliche Überforderung bzw. Spannungen innerhalb der Familie zählten. Einen erhöhten Schweregrad der Verletzungen bei den während des Lockdowns vorgestellten Kinderschutzfällen beobachteten 10 % der teilnehmenden Kliniken und Ambulanzen (*n* = 8). Von 35 % der Einrichtungen (*n* = 27) wurde zurückgemeldet, dass sich infektionsprophylaktische Maßnahmen auf die Anzahl der Kinderschutzfälle ausgewirkt hatten. Beispielsweise war in 9 % der Institutionen (*n* = 7) der Zugang eingeschränkt.

Rund 18 % der Einrichtungen (*n* = 14) wünschten sich für den Kinderschutz während eines pandemiebedingten Lockdowns eine besser abgestimmte Zusammenarbeit mit den Jugendämtern, da in den Einrichtungen behandelte Patienten nicht vom Jugendamt betreut wurden (*n* = 7) oder die Jugendämter schlecht erreichbar waren (*n* = 5). Etwa 13 % der Einrichtungen (*n* = 10) empfahlen, die aufsuchende und ambulante Jugend- bzw. Familienhilfe früher als während des Lockdowns 2020 wieder aufzunehmen. Rund 10 % der Einrichtungen (*n* = 8) sprachen sich dafür aus, Betreuungsangebote für Kinder durch eine frühere und längere Öffnung sowie durch eine Ausweitung der Notbetreuung zu verbessern.

## Diskussion

Die vorliegende Untersuchung auf der Grundlage einer Vollerhebung in kinderversorgenden Kliniken und Ambulanzen in Deutschland beschreibt einen Rückgang der dokumentierten Fallzahlen im medizinischen Kinderschutz während des Lockdowns im Frühjahr 2020 im Vergleich zu 2019. Die Befunde stehen im Einklang mit dem Rückgang an Gefährdungsmeldungen bei den Jugendämtern [16]. Die Ergebnisse der Befragung stützen die von KinderschutzexpertInnen geäußerte Befürchtung, dass von Gewalt betroffene Kinder und Jugendliche während des pandemiebedingten Lockdowns im Frühjahr 2020 seltener erkannt wurden als vor dem Lockdown und lassen sich möglicherweise auf den Verlust von Meldern in Kitas, Schulen und dem außerfamiliären Umfeld aufgrund des „social distancing“ zurückzuführen. Auch infektionsprophylaktische Maßnahmen in den kinderversorgenden Kliniken und Ambulanzen können die Reduktion der Fallzahlen bedingt haben.

Es ist nicht auszuschließen, dass durch geringere Belastungen in der Schule und am Arbeitsplatz das Stressniveau während des Lockdowns für Kinder und Eltern in einigen Familien gesunken ist und dadurch das Misshandlungsrisiko verringert wurde. Allerdings deuten Befragungen zum COVID-19-Lockdown auf erhöhten Stress für Kinder und Erwachsene hin, z. B. durch Überforderung, finanzielle Sorgen, die schwere Vereinbarkeit von Kinderbetreuung und Homeoffice sowie durch die Auflösung eingespielter familiärer Abläufe [15]. Steinert et al. [23] belegten zudem hohe Raten an häuslicher Gewalt während des Lockdowns gegen Frauen und Kinder. Auch die häusliche Gewalt zwischen Eltern stellt im Übrigen eine Kindeswohlgefährdung dar [8] und muss zu den gewonnenen Erkenntnissen zu Kinderschutzfällen während des Pandemie-Lockdowns additiv verstanden werden.

Über die Schwere der Kinderschutzfälle während des Lockdowns können auf der Grundlage der erhobenen Daten keine eindeutigen Aussagen getroffen werden. Einzelne Institutionen meldeten für den Zeitraum eine erhöhte Rate an Fällen mit schweren Verletzungen zurück. Vor dem Hintergrund generell gesunkener Patientenzahlen auch bei sehr schweren Erkrankungen [19] ist zu befürchten, dass die Anzahl an Kinderschutzfällen mit schwerer Misshandlung in diesem Zeitraum mindestens gleich geblieben ist und weniger betroffene Kinder Zugang zu (medizinischer) Hilfe bekommen haben als vor der COVID-19-Pandemie. Diese Vermutung spiegelt sich jedoch nicht in den Daten der Institutionen, die für die Vergleichszeiträume Fallzahlen angegeben habe, wider.

### Limitationen

Die Aussagekraft der Ergebnisse ist durch die unterschiedlichen Standards der in den Kliniken und Ambulanzen dokumentierten Daten sowie durch die unterschiedlichen Definitionen für Fälle und Misshandlungsformen limitiert. Für die Verbesserung der Qualität und Auswertbarkeit der Daten zu Fällen im medizinischen Kinderschutz sollten Standards zur einheitlichen Dokumentation und Definitionen entwickelt werden [13].

Die identifizierten Veränderungen der Fallzahlen lassen sich von jahreszeitlichen oder auf die Einrichtungsgröße zurückgehenden Schwankungen nicht mit Sicherheit abgrenzen. In die Stichprobe waren jedoch viele große Kinderschutzeinrichtungen mit relativ hohen Fallzahlen eingeschlossen (Tab. 1), in denen Schwankungen über die Jahre hinweg als wenig wahrscheinlich angenommen werden können, sodass von einem höchstens geringen Effekt auf die Veränderungen auszugehen ist.

In der Erhebung wurden Fallzahlen aus den ersten 2 Märzwochen 2020 erfasst, in denen die beschränkenden Maßnahmen des Lockdowns noch nicht in Kraft getreten waren. Die erfassten Fallzahlen müssen zudem als Verdachtsfälle betrachtet werden, denen im Rahmen des ambulanten oder stationären medizinischen Kinderschutzes nachgegangen wurde, und können daher nicht als bestätigte Fälle angesehen werden. In Betracht gezogen werden muss, dass die mediale Präsenz des Themas Kinderschutz im Frühjahr 2020 zu einer verstärkten Wahrnehmung möglicher Verdachtsfälle geführt haben könnte. Gegen diese systematische Wahrnehmungsverzerrung spricht jedoch, dass kein Anstieg an Kinderschutzfällen gefunden wurde. Die Fortführung der Datenerhebung zur Entwicklung der Fallzahlen nach dem Lockdown wird dazu weitere Erkenntnisse liefern und auch eine Bewertung der langfristigen Auswirkungen pandemiebedingter Einschränkungen ermöglichen.

### Ausblick

Seit der Erhebung dieser Daten sind längst erneut weitreichende Maßnahmen zur Eindämmung von COVID-19 beschlossen worden, die auch Kinder und Jugendliche betreffen. Langfristige psychologische, wirtschaftliche und gesellschaftliche Folgen der Maßnahmen sind wahrscheinlich, in Art und Umfang aber noch schwierig abzuschätzen. Da die gesundheitlichen und sozioökonomischen Auswirkungen von Gewalt an Kindern weitreichend sind, sollte der Kinderschutz in zukünftigen Maßnahmen zur Eindämmung der COVID-19-Pandemie noch stärker berücksichtigt werden [10, 12]. Kitas und Schulen sollten während eines Lockdowns soweit wie möglich geöffnet bleiben, damit Kinder weiterhin Ansprechpartner außerhalb ihrer Familien haben. Die Kinder- und Jugendhilfe sollte als systemrelevant betrachtet und in Krisenzeiten tätig bleiben. Das Gesundheitswesen sollte Maßnahmen ergreifen, damit Kinder bei einem Verdacht auf Kindeswohlgefährdung infektionssicher untersucht werden können.

## Fazit für die Praxis

- Vorbehaltlich der Limitationen der Studie sind die Fallzahlen im medizinischen Kinderschutz während des Lockdowns in März und April 2020 gegenüber dem Vorjahr deutlich gesunken.
- Es ist zu befürchten, dass das Dunkelfeld misshandelter, missbrauchter und vernachlässigter Kinder während des Lockdowns weiter gestiegen ist.
- Politische und infektionsprophylaktische Maßnahmen müssen v. a. belastete und benachteiligte Familien stärker als zuvor berücksichtigen.
- Die Institutionen der Jugendhilfe sollten in Krisenzeiten als systemrelevant eingestuft werden.
- Falldefinitionen und Dokumentationsstandards im medizinischen Kinderschutz sollten dringend vereinheitlicht werden, um die Situation von Gewalt betroffener Kinder in Deutschland besser bewerten zu können.

## References

[1] Bach I (2020) Familienministerium will Förderung der Kinderschutzhotline verlängern. https://www.tagesspiegel.de/berlin/beratungsangebot-fuer-aerzte-und-therapeuten-familienministerium-will-foerderung-der-kinderschutzhotline-verlaengern/26094748.html. Zugegriffen: 5. Sept. 2020

[2] Berger RP, Fromkin JB, Stutz H (2011). Abusive head trauma during a time of increased unemployment: a multicenter analysis. Pediatrics.

[3] Boserup B, McKenney M, Elkbuli A (2020). Alarming trends in US domestic violence during the COVID-19 pandemic. Am J Emerg Med.

[4] Clemens V, Berthold O, Fegert JM, Kölch M (2018). Kinder psychisch erkrankter Eltern. Nervenarzt.

[5] Clemens V, Deschamps P, Fegert JM (2020). Potential effects of “social” distancing measures and school lockdown on child and adolescent mental health. Eur Child Adolesc Psychiatry.

[6] Curtis T, Miller BC, Berry EH (2000). Changes in reports and incidence of child abuse following natural disasters. Child Abuse Negl..

[7] Fegert JM, Berthold O, Clemens V, Kölch M (2020). COVID-19-Pandemie: Kinderschutz ist systemrelevant. Dtsch Arztebl Int.

[8] Finkelhor D, Turner H, Ormrod R, Hamby SL (2009). Violence, abuse, and crime exposure in a national sample of children and youth. Pediatrics.

[9] Green P (2020). Risks to children and young people during covid-19 pandemic. BMJ.

[10] Habetha S, Bleich S, Weidenhammer J, Fegert JM (2012). A prevalence-based approach to societal costs occurring in consequence of child abuse and neglect. Child Adolesc Psychiatry Ment Health.

[11] Huang MI, O’Riordan MA, Fitzenrider E (2011). Increased incidence of nonaccidental head trauma in infants associated with the economic recession. J Neurosurg Pediatr.

[12] Hughes K, Bellis MA, Hardcastle KA (2017). The effect of multiple adverse childhood experiences on health: a systematic review and meta-analysis. Lancet Public Health.

[13] Jud A, Voll P (2019). The definitions are legion: academic views and practice perspectives on violence against children. Sociological studies of children and youth.

[14] Kofman YB, Garfin DR (2020). Home is not always a haven: the domestic violence crisis amid the COVID-19 pandemic. Psychol Trauma Theory Res Pract Policy.

[15] Langmeyer A, Guglhör-Rudan A, Naab T (2020). Kindsein in Zeiten von Corona.

[16] Mairhofer A, Peucker C, Pluto L (2020). Kinder- und Jugendhilfe in Zeiten der Corona-Pandemie.

[17] Morand A, Fabre A, Minodier P (2020). COVID-19 virus and children: what do we know?. Arch De Pédiatrie.

[18] Piquero AR, Riddell JR, Bishopp SA (2020). Staying home, staying safe? A short-term analysis of COVID-19 on dallas domestic violence. Am J Crim Just.

[19] Ramshorn-Zimmer A, Schröder R, Gries (2020). Notaufnahme während der Coronapandemie: Weniger Non-COVID-19-Notfälle. Dtsch Arztebl.

[20] Schwier F, Manjgo P, Kieslich M (2019). Neue Entwicklungen im medizinischen Kinderschutz. Monatsschr Kinderheilkd.

[21] Sidpra J, Abomeli D, Hameed B (2020). Rise in the incidence of abusive head trauma during the COVID-19 pandemic. Arch Dis Child.

[22] Statistisches Bundesamt (2020). Gefährdungseinschätzungen nach § 8a Absatz 1 SGB VIII - 2019.

[23] Steinert J, Ebert C (2020) Gewalt an Frauen und Kindern in Deutschland während COVID-19-bedingten Ausgangsbeschränkungen: Zusammenfassung der Ergebnisse

[24] Winter H (2020) Viele psychische Krankheiten können durch die Pandemie forciert werden. Dtsch Aerztebl. https://www.aerzteblatt.de/nachrichten/111097/Viele-psychische-Krankheiten-koennen-durch-die-Pandemie-forciert-werden. Zugegriffen: 15.02.2020

[25] Wood JN, French B, Fromkin J (2016). Association of pediatric abusive head trauma rates with macroeconomic indicators. Acad Pediatr.

[26] Zitelmann M, Berneiser C, Beckmann K (2020). Appell aus der Wissenschaft: Mehr Kinderschutz in der Corona-Pandemie.

[27] Nummer gegen Kummer (2020) Corona und die aktuelle Situation bei „Nummer gegen Kummer“. https://www.nummergegenkummer.de/neues/corona-und-die-aktuelle-situation-bei-nummer-gegen-kummer.html. Zugegriffen: 5. Sept. 2020

